# Targeted UHPLC–HRMS (Orbitrap) Polyphenolic and Capsaicinoid Profiling for the Chemometric Characterization and Classification of Paprika with Protected Designation of Origin (PDO) Attributes

**DOI:** 10.3390/molecules25071623

**Published:** 2020-04-01

**Authors:** Sergio Barbosa, Javier Saurina, Lluís Puignou, Oscar Núñez

**Affiliations:** 1 Department of Chemical Engineering and Analytical Chemistry, University of Barcelona, E08028 Barcelona, Spain; sergiobarbosabarbero@hotmail.com (S.B.); xavi.saurina@ub.edu (J.S.); lluis.puignou@ub.edu (L.P.); 2 Research Institute in Food Nutrition and Food Safety, University of Barcelona, E08921 Barcelona, Spain; 3 Serra Húnter Fellow, Generalitat de Catalunya, E08007 Barcelona, Spain

**Keywords:** targeted analysis, UHPLC, high-resolution mass spectrometry, paprika, protected designation of origin (PDO), polyphenols, capsaicinoids, food authentication, chemometrics

## Abstract

Society’s interest in the quality of food products with certain attributes has increased, the attribute of a Protected Designation of Origin (PDO) being an effective tool to guarantee the quality and geographical origin of a given food product. In Spain, two paprika production areas with PDO (La Vera and Murcia) are recognized. In the present work, targeted UHPLC-HRMS polyphenolic and capsaicinoid profiling through the TraceFinder^TM^ screening software, using homemade accurate mass databases, was proposed as a source of chemical descriptors, to address the characterization, classification, and authentication of paprika. A total of 126 paprika samples from different production regions—Spain (La Vera PDO and Murcia PDO) and the Czech Republic, each including different flavor varieties, were analyzed. UHPLC-HRMS polyphenolic profiles showed to be good chemical descriptors to achieve paprika classification and authentication, based on the production region, through principal component analysis and partial least squares regression-discriminant analysis, with classification rates of 82%, 86%, and 100% for La Vera PDO, Murcia PDO, and the Czech Republic, respectively. In addition, a perfect classification was also accomplished among the flavor varieties for the Murcia PDO and Czech Republic samples. By employing the UHPLC-HRMS polyphenolic and capsaicinoid profiles as chemical descriptors, acceptable discrimination among La Vera PDO flavor varieties was also achieved.

## 1. Introduction

Paprika is a red powder spice with a very characteristic flavor obtained after the drying and grinding of certain varieties of red peppers of the genus *Capsicum*, which belongs to the Solanaceae family [1]. Within this genus, there are approximately 39 species, such as *C. annuum*, *C. chinense*, *C. baccatum*, *C. frutescens*, and *C. pubescens*, grown in different parts of the world, including wild, semi-domestic, and domestic ones; *Capsicum annuum* is the most common among these [1,2]. Paprika spice is commonly used to add flavor and color to many foods, such as baked goods, beverages, meat, soups, ice creams, candies, and seasoning mixes, but other applications in medicine, cosmetics, personal protection sprays, or even as adsorbents for the removal of contaminants, are reported [3,4,5,6].

Nowadays, food manufacturers, as well as the general public, are increasingly concerned about the quality of food with certain attributes, and as a consequence, the demand for food products of a specific geographical origin has increased. Within this context, the attribute of a Protected Designation of Origin (PDO) is an effective tool to guarantee the quality and geographical origin of a given food product. According to the European Union Council Regulation (EC) No. 510/2006 [7], PDO establishes the name of a region, of a specific place or, in exceptional cases, of a country that is used to designate an agricultural product or a food product; which must originate from an established region, a specific place, or a country; whose quality or characteristics are fundamentally or exclusively a result of the geographical environment, including its natural and human factors; and whose production, transformation, and elaboration are carried out in the defined geographical area.

In Spain, there are two paprika production areas with PDO attributes recognized by the European Union—the region of La Vera in Cáceres (Extremadura) and the region of Murcia [8,9,10]. Despite having a common origin and an almost parallel development, the production process is different in each of these areas [11]. In both cases, the product is the result of the drying and grinding of pepper fruits from the *Capsicum* genus, but the drying process and the fruit varieties change, which also provides specific organoleptic characteristics. Thereby, the red peppers used for the production of “Pimentón de la Vera” belong to the varieties of Ocales (Jaranda, Jariza, and Jeromín) and Bola. Three different flavor varieties are produced, depending on the type of red peppers employed—sweet, spicy, and bittersweet paprika. Additionally, La Vera paprika is characterized by its smoky aroma and taste achieved during the drying process using smoke produced with oak or holm oak wood. In contrast, pepper fruits used for the Murcia PDO paprika belong mainly to the Bola variety, which is sweet and lightweight, and is dried under the sun.

Paprika is characterized by containing a large number of bioactive compounds with great beneficial health properties, such as carotenoids (provitamins), ascorbic acid (vitamin C), tocopherols (vitamin E), capsaicinoids, and phenolic compounds [12]. Among them, the importance of polyphenolic and phenolic compounds that are widely distributed in plants is worth noting, many of which are essential metabolites that contribute to the sensory properties associated with the quality of foods, such as color and aroma [13]. These compounds have a strong antioxidant activity and have shown potential health benefits, such as vascular protection, antihepatotoxic, antiallergic, antiproliferative, antiosteosporotic, anti-inflammatory, antitumor, antidiabetic, antiobesity, etc. [13,14,15,16,17,18]. Previous studies have reported that the main phenolic compounds found in paprika are vanillic, caffeic, ferulic, *p*-coumaric, and *p*-hydroxybenzoic acids [18]. The presence of capsaicinoids is also very characteristic in paprika samples and is responsible for their pungency flavor [19,20,21,22]. In general, the pungency of the *Capsicum* species depends on the concentration of capsaicinoids, particularly capsaicin, in the pepper fruit. Usually, sweet paprika has very low capsaicinoid contents and their level increases in the spicy paprika.

At present, there are several methods for the determination of polyphenolic and phenolic compounds, such as global assays for the evaluation of the total content by colorimetric or fluorimetric methods, or more specific ones, based on separation by capillary electrophoresis, liquid chromatography, and gas chromatography. However, liquid chromatography with UV or electrochemical detection, or that coupled with mass spectrometry is nowadays the technique of choice [17,23,24,25,26,27,28,29,30,31,32]. However, the great chemical diversity among this family of compounds and the range of concentrations in which they can be found make liquid chromatography coupled with high-resolution mass spectrometry (LC-HRMS) the most effective technique. Among these, time-of-flight (TOF) and Orbitrap analyzers are the most frequently employed for the characterization, identification, and determination of polyphenolic and phenolic compounds in food, including paprika products [25,27,31]. Liquid chromatography with UV and mass spectrometric detection are also typically employed in the determination of capsaicinoid compounds in pepper and pepper-processed products [14,33,34,35,36,37,38,39].

It is worth noting that the content of paprika bioactive substances, such as polyphenols and capsaicinoids might differ due to multiple parameters, such as the pepper variety, the climate conditions, growing areas, water resources, ripening stage, agronomy conditions, pre- and postharvest treatments, etc. [13]. As a result, polyphenolic and capsaicinoid distribution and content profiling in a food product might be proposed as a source of analytical data to establish sample classification and authentication, for both the correct assignment of the product PDO attributes and the prevention of fraud practices through product adulteration. Paprika is a worldwide consumed spice so its adulteration might offer high economic benefits. Within this context, the substitution of ingredients, the addition of (illegal) substances and false declaration of origin are important and challenging issues to the authorities and the food industry. Both targeted and non-targeted (fingerprinting) methodologies—some in combination with multivariate chemometric methods such as principal component analysis (PCA) and partial least squares regression-discriminant analysis (PLS-DA) have been proposed to address paprika classification and authentication issues [13,29,39,40,41,42,43,44,45,46]. As an example, we recently proposed a UHPLC-MS/MS method, using a triple quadrupole instrument for the determination of 36 polyphenols and phenolic compounds in paprika samples, which was applied to PDO authentication by PCA and PLS-DA [28]. Although in general a good sample discrimination regarding the production region was observed, the targeted monitoring of these 36 compounds was not good enough to achieve the discrimination of La Vera Paprika samples as a function of the flavor variety (sweet, bittersweet, and spicy).

This work aims for the application of a targeted-UHPLC-HRMS method using an Orbitrap analyzer for polyphenolic and capsaicinoid profiling, and the use of the obtained profiles as sample chemical descriptors, to address the classification and authentication of paprika samples as a function of both production regions and flavor varieties. For this purpose, a total of 126 paprika samples belonging to La Vera PDO, Murcia PDO (Spain), and the Czech Republic were analyzed through the proposed methodology, after applying a simple sample extraction procedure utilizing water:acetonitrile 20:80 (*v*/*v*) as an extractant agent. As a first approach, 53 polyphenols and phenolic compounds were monitored using the TraceFinder^TM^ software v3.3 (Thermo Fisher Scientific, San José, CA, USA), employing a previously established accurate mass database [27], and the obtained peak signal profiles were used as sample chemical descriptors for PCA and PLS-DA. Then, to improve sample classification, especially for the La Vera paprika samples, capsaicinoid profiling by monitoring 12 compounds was also employed as sample chemical descriptors, together with the polyphenolic and phenolic acid profiles, for PCA and PLS-DA.

## 2. Results and Discussion

### 2.1. Targeted UHPLC–HRMS Polyphenolic Profiling

**Figure 1:**
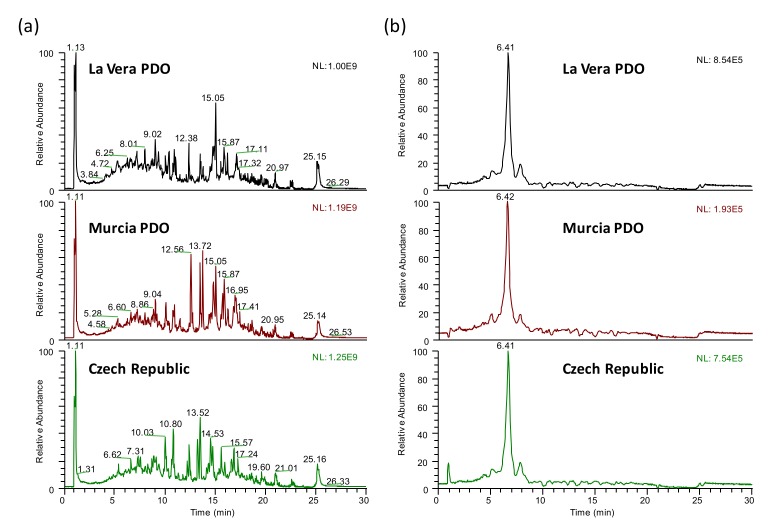
Ultra-high performance liquid chromatography-high-resolution mass spectrometry (UHPLC-HRMS) total ion chromatograms (**a**) and the extracted ion chromatograms for ferulic acid (*m*/*z* 193.0506) (**b**) of the three selected sweet La Vera Protected Designation of Origin (PDO), Murcia PDO, and Czech Republic paprika samples.

As previously commented, the present work aimed to develop a targeted-UHPLC-HRMS profiling method to obtain discriminant paprika chemical descriptors that able to address sample classification regarding both the paprika production region (and PDO) and the paprika flavor variety. For this purpose, the paprika samples were analyzed through reversed-phase chromatography using a C18 column under universal gradient elution, with water and acetonitrile (both with 0.1% formic acid) as the mobile phase components, after a simple sample extraction with water:acetonitrile 20:80 (*v*/*v*), as previously reported [28,29,46]. As an example, Figure 1 shows the UHPLC–HRMS total ion chromatograms and the selected ion chromatogram for the ferulic acid (*m*/*z* 193.0506) of three selected sweet paprika samples belonging to different production regions (La Vera PDO, Murcia PDO, and the Czech Republic). As can be seen, remarkable differences in both signal profile and relative abundances are obtained, depending on the origin of the samples. As a first approach, chromatographic sample raw data was then processed with the TraceFinder^TM^ software (Thermo Fisher Scientific) to obtain the corresponding targeted-UHPLC-HRMS polyphenolic profiles, by using an accurate mass database of 53 polyphenols and phenolic compounds that were previously characterized [27]. A threshold signal of 1.0 × 10^5^ was established in the screening software to consider a positive match for a given polyphenolic compound in the analyzed samples. In addition, compound confirmation was only granted if all the above-mentioned confirmation criteria (retention time, accurate mass measurement errors lower than 5 ppm, isotopic pattern agreement higher than 85%, and product ion spectra) were accomplished. After raw data processing with the TraceFinder^TM^ screening software (Thermo Fisher Scientific), a report was provided for each analyzed sample, depicting the peak areas of all polyphenolic compounds detected and confirmed (as an example, Table 1 shows the TraceFinder^TM^ (Thermo Fisher Scientific) report obtained for the La Vera PDO sweet paprika sample). UHPLC-HRMS polyphenolic profiles consisting of the peak areas extracted by the TraceFinder^TM^ software (Thermo Fisher Scientific) in the analyzed paprika and quality control (QC) samples were then obtained.

### 2.2. Sample Exploration by PCA

**Figure 2:**
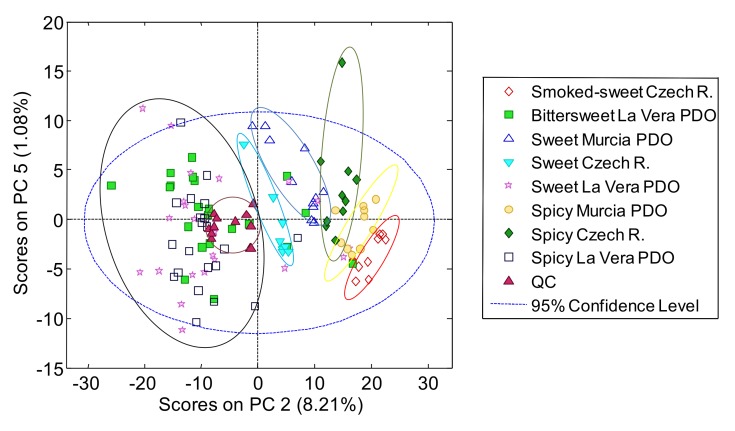
Principal component analysis (PCA) score plot of PC2 vs. PC5 when using the UHPLC-HRMS polyphenolic profiles as paprika sample chemical descriptors.

A data matrix containing the peak areas of the UHPLC-HRMS polyphenolic profiles of all analyzed paprika and QC samples was built to the non-supervised PCA exploration. The dimension of this data matrix was 140 samples and QCs × 53 variables. Data were normalized for each compound with respect to the overall analyte signal, to provide similar weighs to all samples. Figure 2 depicts the best PCA score plot (PC2 vs. PC5) obtained. A total of 5 PCs were required for the construction of the PCA model.

As can be seen in Figure 2, the QCs appeared grouped, showing a good performance of the proposed methodology and the robustness of the obtained PCA chemometric results. QCs were also grouped close to the center area of the plot, although within the region where the La Vera paprika samples were clustered, as the QCs were enriched on the La Vera paprika sample attributes and this was the group with the largest number of samples. Regarding paprika sample distribution, almost all La Vera PDO samples had negative scores on PC2 (being located at the left part of the plot), although no discrimination among flavor varieties (sweet, bittersweet, and spicy) was observed. In contrast, the Murcia PDO and the Czech Republic paprika samples had positive PC2 scores (all of them located in the right area of the score plot). In addition, the samples were more or less distinguished in terms of both the production region and the flavor varieties. The sweet Czech Republic samples were closest to the La Vera samples, followed by the sweet Murcia PDO, spicy Czech Republic, spicy Murcia PDO, and finally the smoked-sweet Czech Republic samples, as the PC2 scores increased.

**Figure 3:**
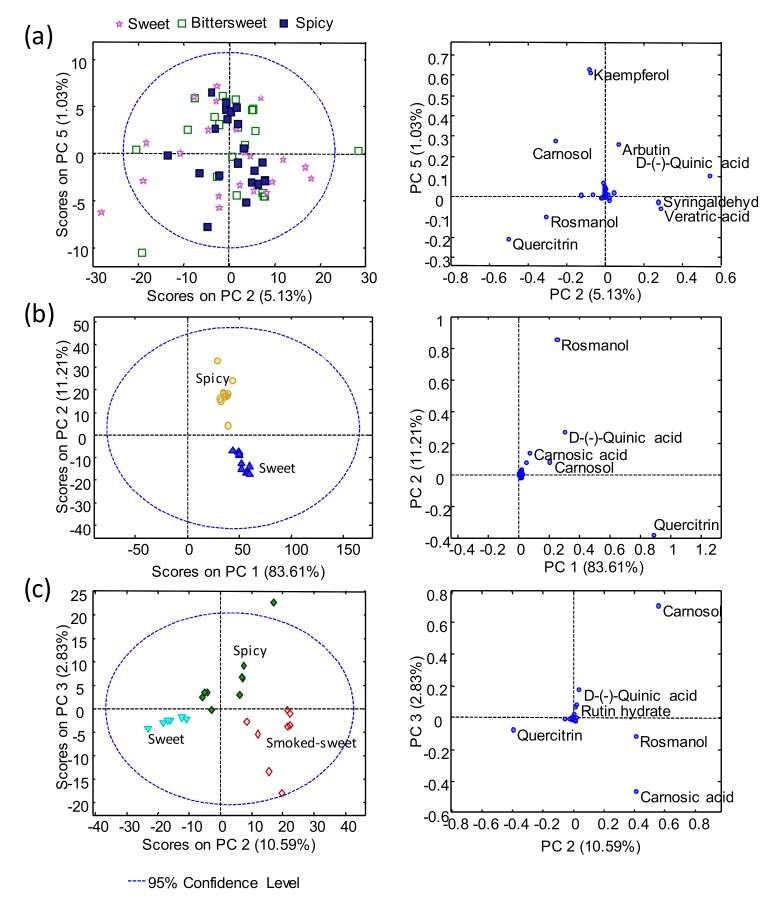
Principal component analysis (PCA) score and loading plots for (**a**) the La Vera PDO paprika samples, (**b**) the Murcia PDO paprika samples, and (**c**) the Czech Republic paprika samples.

PCA models for each paprika production region were also independently evaluated, to study the distribution of samples according to each paprika flavor varieties, and the best score and loading plots obtained are depicted in Figure 3.

These models enhanced the fact that no discrimination was accomplished among the La Vera paprika samples, regarding their three different flavor varieties (sweet, bittersweet, and spicy), when using the UHPLC-HRMS polyphenolic profiling of the 53 studied compounds, as chemical descriptors for PCA (Figure 3a). In contrast, as expected, the flavor varieties were perfectly discriminated for both Murcia PDO and Czech Republic paprika samples. In the case of the Murcia PDO samples (Figure 3b), the spicy ones were located at the top of the PCA plot, with rosmanol being the most discriminant compound for this group of samples. Whereas, quercitrin was the most discriminant compound for the sweet flavor variety, which were clustered at the plot, exhibiting negative PC2 scores. For the Czech Republic samples, both PC2 and PC3 allowed a perfect separation of the three different flavor varieties under study. The three groups were mainly separated by PC2, with the sweet samples exhibiting negative scores, the spicy samples being located in the middle, and the smoked-sweet samples showing positive PC2 scores. PC3 was related to the spicy attribute, being the only group exhibiting clearly positive PC3 score values. Regarding the polyphenolic compounds employed to achieve this sample discrimination, it seemed that quercitrin contributed to the sweet flavor variety, while carnosol and carnosic acid were clearly responsible for the discrimination of the spicy and smoked-sweet flavor varieties, respectively. It is well-known that the type of polyphenols and their content in plant products depends on multiple factors, such as the climatic conditions of the production region. One possible reason why the polyphenols analyzed in this work did not allow a discrimination between the different varieties of paprika from La Vera PDO was due to the fact that it is a very small region, with only 1282 hectares of production, compared to Murcia, for example, with more than 220,000 hectares of production, which meant that the climatic conditions in the La Vera region were very similar in the production of its three flavor varieties.

### 2.3. Sample Classification by PLS-DA

**Figure 4:**
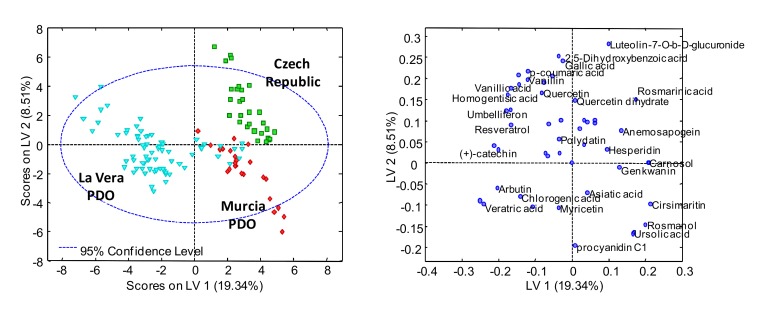
Partial least squares regression-discriminant analysis (PLS-DA) score and the loading plots of LV1 vs. LV2 for the classification of the analyzed paprika samples, according to their PDO and production region.

A supervised classificatory chemometric analysis of the paprika samples was done by employing PLS-DA. Thus, while the X-data matrix was similar to that used in the PCA without the QCs, the Y-data matrix indicated the membership of each paprika sample. A total of 4 LVs were required for the construction of the PLS-DA model. Figure 4 shows the PLS-DA score and the loading plots obtained when the UHPLC-HRMS polyphenolic profiles were used as chemical descriptors, to address the classification of the paprika samples through the PDO and production region. As can be seen, a good sample classification was obtained, with the La Vera PDO samples being located on the left-side of the plot, while the Murcia PDO and the Czech Republic samples were located on the right. These last two groups of samples were mainly discriminated by LV2, with the Murcia PDO samples displaying negative values of LV2, while the Czech Republic samples showed positive values. It should be mentioned that several La Vera PDO and Murcia PDO samples were mixed, although the Spanish paprika samples were completely discriminated from those produced in the Czech Republic. The proposed UHPLC-HRMS polyphenolic profiling method of monitoring 53 polyphenolic and phenolic acids seemed to be appropriate to address paprika classification and authentication, according to their PDO and production region.

The study of the PLS-DA loading plot allowed several phenolic and polyphenolic compounds to be identified, such as vanillic acid, homogentisic acid, umbelliferon, resveratrol, (+)-catechin, arbutin, chlorogenic acid, and veratric acid, which contributed to the separation of the La Vera PDO paprika samples from the other paprikas. Ursolic acid, rosmanol, and cirsimaritin seemed to be the most discriminant variables for the classification of the Murcia PDO samples, while the presence of luteolin-7-O-β-D-glucuronide, 2,5-dihydroxybenzoic acid, gallic acid, and rosmarinic acid seemed to be enhanced in the Czech Republic samples.

A 3D PLS-DA model was also built to classify the analyzed samples, according to both production region and flavor variety; the obtained results are shown in Figure S1 (Supplementary Materials). This model showed that the discrimination of the three paprika production regions was fully accomplished and was able to observe a perfect separation of both Murcia PDO and La Vera PDO groups, in comparison to the plot in Figure 4. In addition, a perfect discrimination was also achieved for the different flavor varieties of both the Murcia PDO and the Czech Republic samples. In contrast, no separation was observed for the three La Vera PDO paprika flavor varieties.

**Figure 5:**
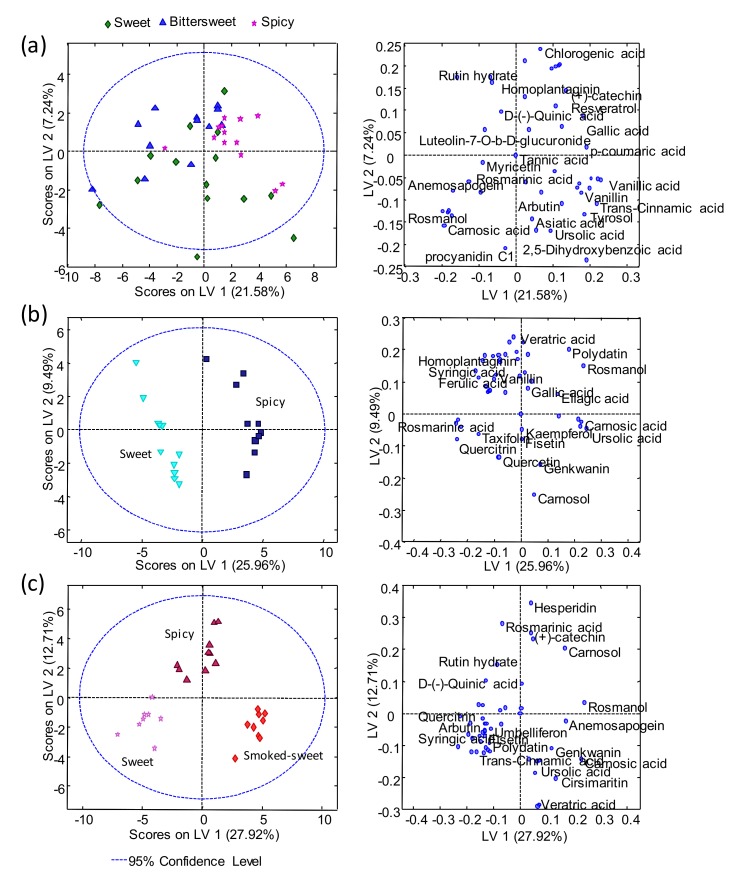
Partial least squares regression-discriminant analysis (PLS-DA) score and loading plots for (**a**) La Vera PDO paprika samples, (**b**) Murcia PDO paprika samples, and (**c**) the Czech Republic paprika samples.

PLS-DA models for each paprika production region was also independently evaluated, to study the distribution of samples according to each paprika flavor varieties, and the best score and loadings plots obtained are depicted in Figure 5.

With regards to the La Vera PDO paprika samples, the PLS-DA results were slightly better than the ones obtained by PCA, as expected when using a classificatory chemometric method. Although not perfectly separated, a tendency in the distribution of the three flavor varieties was observed. The sweet variety tended to be distributed at the bottom of the plot, exhibiting negative LV2 values, while the bittersweet and spicy flavors tended to be distributed to the top-left and the top-right areas of the plot, respectively. Considering the loadings plot (Figure 5a), procyanidin C1, 2,5-dihydroxybenzoic, and carnosic and ursolic acids seemed to be the most discriminant compounds for the sweet La Vera PDO samples; resveratrol, gallic acid, and *p*-coumaric acid were the most discriminant for the spicy La Vera PDO samples; and rutin hydrate and luteolin-7-O-β-D-glucuronide were the compounds that were more discriminant for the bittersweet La Vera PDO samples. In relation to the other two groups of paprika samples, sample classification by PLS-DA according to the flavor varieties was perfectly accomplished. Polydatin, rosmanol, ellagic acid, carnosic acid, ursolic acid, genkwanin, and carnosol were among the polyphenolic and phenolic compounds responsible for the discrimination of Murcia PDO spicy paprika (Figure 5b). While rosmarinic acid, quercitrin, homoplantaginin, syringic acid, and ferulic acid were the ones responsible for the discrimination of the Murcia PDO sweet variety. In the case of the Czech Republic samples, hesperidin, rosmarinic acid, (+)-catechin, rutin, and carnosol explained the discrimination of the spicy variety; ursolic acid, cirsimaritin, carnosic acid, and veratric acid were among the polyphenolic compounds allowing the discrimination of the smoked-sweet variety; and finally, arbutin, syringic acid, and trans-cinnamic acid were among the ones allowing for the discrimination of the sweet variety.

Up to this point, it seemed that the proposed UHPLC-HRMS polyphenolic profiling was a good method to obtain discriminant sample chemical descriptors to address the classification of the analyzed paprika samples as a function of the PDO and production region, and also that of the flavor variety, with the exception of the La Vera PDO samples where only a tendency was observed.

### 2.4. Supervised PLS-DA Method Validation

To demonstrate the applicability of the proposed methodology based on the UHPLC–HRMS polyphenolic profiling, the classification rate was studied for some paired PLS–DA models: (i) La Vera PDO vs. the other samples, (ii) Murcia PDO vs. the other samples, and (iii) the Czech Republic vs. the other samples. For this purpose, the PLS-DA model was built with a calibration set composed of 70% of the samples belonging to each class, while the other 30% of the samples were used as the test set for prediction purposes.

**Figure 6:**
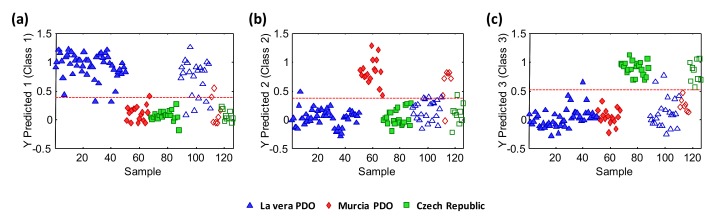
Partial least squares regression-discriminant analysis (PLS-DA) classification plots according to the production region. (**a**) La Vera PDO vs. other classes; (**b**) Murcia PDO vs. other classes; (**c**) the Czech Republic vs. other classes. Sample assignment: triangle = La Vera, rhombus = Murcia, and square = Czech Republic. Filled symbols = calibration set, empty symbols = validation/prediction set. The dashed line means the classification boundary.

Figure 6 shows the classification plots obtained. The dashed line indicates the classification boundary, so the samples belonging to the targeted class were located at the top, while those belonging to the other types were located at the bottom. Samples to be used for calibration were on the left (with filled symbols) and those used for prediction were on the right side (with empty symbols).

As can be seen, acceptable classification results were obtained. When targeting the La Vera PDO samples vs. all the other samples (Figure 6a), 82% of the samples were correctly predicted (only 4 samples of the 22 randomly selected for prediction were not correctly classified as belonging to the La Vera PDO samples). This was expected, as a complete discrimination between the La Vera PDO and the Murcia PDO was not fully achieved, as was described in Figure 4. Nevertheless, taking into account the type of samples and their variability, this classification rate could be considered to be very acceptable. The classification rates noticeably improved when addressing the classification of the Murcia PDO samples (Figure 6b) and the Czech Republic samples (Figure 6c), with respect to the other sample groups. Thus, only one Murcia PDO sample was not correctly classified out of the 7 samples that were randomly selected for prediction (classification rate of 86%), while all 9 Czech Republic samples were correctly classified (100% classification rate). The confusion matrix was [18, 2, 2; 1, 6, 0; 0, 0, 9] for La Vera PDO, Murcia PDO, and Czech Republic, respectively.

### 2.5. Targeted UHPLC-HRMS Polyphenolic and Capsaicinoid Profiling

As previously discussed, UHPLC-HRMS polyphenolic profiling resulted in good chemical descriptors to address the paprika sample classification and authentication regarding the paprika PDO and production region, and also according to the different flavor varieties commercialized, for both the Murcia PDO and Czech Republic paprika. However, only a slight tendency was achieved in the discrimination of the three La Vera PDO paprika varieties (Figure 5a). With the aim of improving this discrimination, the targeted profiling of other bioactive compounds that are typically present in paprika samples, such as capsaicinoids and capsinoids, was performed. For this purpose, twelve capsaicinoid and capsinoid compounds (Table S1) that are typically characteristic of spicy pepper were selected. As pure standards were not commercially available for all selected compounds, in the present work, the targeted screening for this family of chemicals was proposed based only on the HRMS spectra accurate mass measurements (Table S2), and with this information, a homemade accurate mass database was built using the TraceFinder^TM^ software (Thermo Fisher Scientific).

Paprika UHPLC-HRMS raw data was then processed with the TraceFinder^TM^ screening software (Thermo Fisher Scientific), with both polyphenolic and capsaicinoids accurate mass databases. Again, a threshold signal of 1.0 × 10^5^ was established in the screening software, to consider a positive match for a given targeted compound in the analyzed samples. Compound confirmation was only granted if all established confirmation criteria were established, i.e., accurate mass measurements lower than 5 ppm, and an isotopic pattern agreement higher than 85% for both profiled families of compounds. Retention times and product ion spectra agreement was also employed for the polyphenolic compounds, as characterization with commercially available standards was previously accomplished. UHPLC-HRMS polyphenolic and capsaicinoid profiles, consisting of peak areas extracted by the TraceFinder^TM^ software (Thermo Fisher Scientific) in the analyzed paprika samples, were then obtained and subjected to PLS-DA.

**Figure 7:**
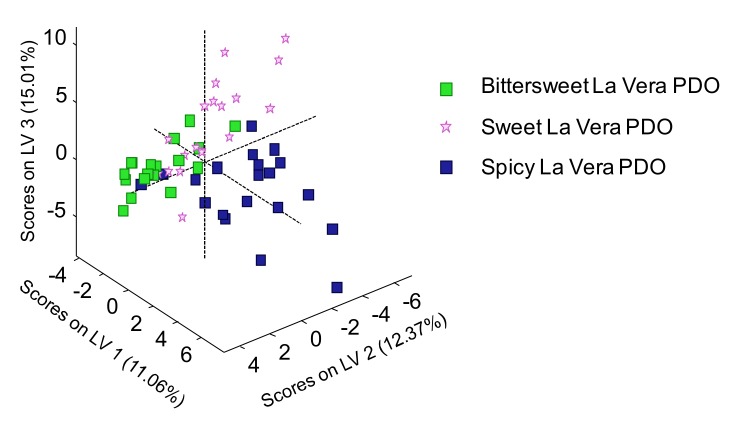
3D plot of scores of PLS-DA (LV1 vs. LV2 vs. LV3) when the UHPLC-HRMS polyphenolic and capsaicinoid profiles were employed as chemical descriptors for the La Vera PDO sample classification, according to the flavor variety.

Focusing only on the La Vera PDO paprika flavor varieties (sweet, bittersweet, and spicy), Figure 7 shows the 3D plot of the scores through PLS-DA (LV1 vs. LV2 vs. LV3), when the obtained UHPLC-HRMS polyphenolic and capsaicinoid profiles were employed as sample chemical descriptors to address the La Vera PDO paprika sample classification. As can be seen, the combination of both polyphenolic and capsaicinoid profiling, clearly improved the classification and discrimination of the three flavor varieties commercially available in the La Vera PDO. The spicy variety was clearly discriminated from the other two flavor varieties, exhibiting positive LV1 values, which was expected due to the high presence of capsaicinoid and capsinoid compounds in spicy paprika products. In addition, better sample discrimination was also observed between the sweet and bittersweet flavor varieties, based mainly on LV2.

## 3. Materials and Methods

### 3.1. Chemicals and Standard Solutions

Unless otherwise stated, all chemicals, standards, and reagents employed in this work were of analytical grade. Fifty-three polyphenolic and phenolic standards belonging to different families (phenolic acids, benzoic acids, cinnamic acids, phenolic aldehydes, phenolic terpenes, flavones, flavanols, proanthocyanidins, and stilbenes) characterized by HRMS in a previous work [27], were obtained from Sigma-Aldrich (Steinheim, Germany).

LC-MS grade water, methanol, acetonitrile, formic acid (98–100%), and acetone were also obtained from Sigma-Aldrich, and hydrochloric acid (37%) was obtained from Merck (Seelze, Germany).

Stock standard solutions of all compounds (∼1000 mg/L) were prepared in methanol in amber glass vials. Intermediate working solutions were prepared weekly from these stock standard solutions through appropriate dilution with water. All stock solutions were kept in the refrigerator at 4 °C, for not more than 1 month.

### 3.2. Instrumentation

Sample analyses were performed using a UHPLC system (Thermo Fisher Scientific, San José, CA, USA) equipped with a quaternary pump, an autosampler, and a column oven. The UHPLC system was coupled to a Q-Exactive Orbitrap HRMS system (Thermo Fisher Scientific), with a heated electrospray ionization source (HESI-II) operating in a negative ionization mode. An Ascentis^®^ Express C18 porous shell reversed-phase column (150 × 2.1 mm, 2.7 µm particle size) provided by Supelco (Bellefonte, PA, USA) was employed for the chromatographic separation. A binary system of solvents, based on a 0.1% formic acid aqueous solution (Solvent A) and a 0.1% formic acid in acetonitrile (Solvent B) was used to establish the elution gradient at a mobile phase flow rate of 0.3 mL/min. The elution gradient employed was as follows: 0–1 min, isocratic conditions at 10% B, 1–20 min, linear gradient from 10% to 95% B; 20–23 min, isocratic step at 95% B; 23–24 min back to initial conditions at 10% B; and from 24 to 30 min, isocratic elution at initial conditions for column re-equilibration. An injection volume of 10 µL (full-loop mode) was employed, and the column was kept at room temperature.

Nitrogen was employed as sheath gas, sweep gas, and auxiliary gas for the HESI-II ionization source at flow rates of 60, 0, and 10 a.u. (arbitrary units), respectively. HESI-II heater temperature and capillary voltage were set at 350 °C and −2.5 kV, respectively. The capillary instrument temperature was maintained at 320 °C, and an S-Lens RF level at 50 V was used. The Orbitrap analyzer was tuned and calibrated every 3 days, by employing a commercially available calibration solution for this purpose (Thermo Fisher Scientific). HRMS spectra were acquired in full MS scan mode with an *m/z* range from 100 to 1500 at a mass resolution of 70,000 full width at half-maximum (FWHM, at *m/z* 200), an automatic gain control (AGC) target (number of ions to fill the C-Trap) of 2.5 × 10^5^, and a maximum injection time (IT) of 200 ms. The full MS scan mode was followed by a data-dependent scan mode operating in a product ion scan, obtained by applying stepped normalized collision energies (NCE) of 17.5, 35, and 52.5 eV, with a fixed first *m*/*z* value of 50 Da for the registered *m*/*z* product ion range. This data-dependent scan mode was activated with an intensity threshold of 1.0 × 10^5^, with a quadrupole isolation window of 0.5 *m*/*z*. At this stage, a mass resolution of 17,500 FWHM (at *m*/*z* 200), an AGC value of 2.0 × 10^5^, and an IT value of 200 ms were employed.

### 3.3. Samples and Sample Treatment

A total of 126 paprika samples purchased from local markets in Spain and the Czech Republic were analyzed. Samples from the Czech Republic were also analyzed in order to demonstrate the applicability of the proposed methodology, to discern the different countries of origin among the samples. Among them, 72 samples were from La Vera PDO (26 sweet, 23 bittersweet, and 23 spicy flavors), 24 samples from the Murcia PDO (12 sweet and 12 spicy flavors), and 30 samples were from the Czech Republic (10 sweet, 10 smoked-sweet, and 10 spicy flavors).

Sample treatment was carried out, following a previously described method [28,29,46]. In brief, 0.3 mg of paprika powder was extracted with 3 mL of water:acetonitrile 20:80 (*v*/*v*) solution, by stirring in a vortex mixer (Stuart, Stone, United Kingdom) for 1 min, and by sonication (2510 Branson ultrasonic bath, Hampton, NH, USA) for 15 min. Then, centrifugation for 30 min at 4500 rpm (Rotana 460 HR centrifuge, Hettich, Germany) was performed, and the supernatant was transferred into a 2 mL glass injection vial, after filtration through 0.45 µm nylon filters (Whatman, Clifton, NJ, USA). The extracts were kept in the freezer at −18 °C, until analysis.

A quality control (QC) solution was prepared by mixing 50 µL of each paprika sample extract. This QC solution was used to evaluate the repeatability of the proposed method and the robustness of the chemometric results.

All paprika samples were analyzed randomly with the proposed UHPLC-HRMS method to prevent the variations caused by the sequence duration. Moreover, a QC and an instrumental chromatographic blank of acetonitrile were also analyzed every 10 paprika samples.

### 3.4. Data Analysis

UHPLC-HRMS raw chromatographic data were processed by the TraceFinder^TM^ v3.3 software (Thermo Fisher Scientific), by applying two user-targeted accurate mass database lists: (i) one list comprised the 53 previously characterized polyphenol and phenolic compounds [27], and (ii) another list comprised 12 capsaicinoid and capsinoids (see Tables S1 and S2 in the Supplementary Materials). Several confirmation criteria such as chromatographic retention times (for all studied polyphenol and phenolic compounds), accurate mass errors (values below 5 ppm), and isotopic pattern (matches higher than 85%) were employed with the TraceFinder^TM^ software (Thermo Fisher Scientific) to assess the presence of the targeted compounds in the analyzed samples.

PCA and PLS-DA chemometric analysis were performed using the Stand Alone Chemometric Software (SOLO) obtained from Eigenvector Research [47]. A theoretical background description regarding these chemometric procedures is described elsewhere [48].

For polyphenolic profiling, X-data matrices to be treated by PCA and PLS-DA consisted of the peak area values of the 53 polyphenol and phenolic acids found and confirmed in the analyzed QCs and the paprika samples, using the TraceFinder^TM^ software (Thermo Fisher Scientific). These polyphenolic profiles were then employed as sample chemical descriptors. The dimension of the data matrix was 126 (samples + QCs) × 53 analyte peak areas. When both, polyphenolic and capsaicinoid profiling was considered, the peak area values of the 12 studied capsaicionids and capsinoids were also incorporated in the data matrix, providing a matrix dimension of 140 (samples + QCs) × 65 analyte peak areas. In all cases, normalization pretreatment concerning the overall analyte concentration was applied to provide similar weights to all samples. Y-data matrix in the PLS–DA models was defined by the membership of each sample in the corresponding class. Scatter plots of scores and loadings from principal components (PCs), when PCA was used and from latent variables (LVs), for PLS-DA, were employed to study the distribution of samples and variables (analyte peak areas). Information regarding correlations and dependences for the targeted compounds with the paprika products analyzed was, thus, visualized. The most appropriate number of LVs was established by considering the first significant minimum point of the cross-validation (CV) error from a Venetian blind approach. Additionally, the applicability of the built chemometric models was proved by validating them on an independent prediction set. In particular, the validation of the PLS-DA models was carried out by using 70% of a sample group as the calibration set, while the remaining 30% constituted the prediction set.

## 4. Conclusions

In the present work, targeted UHPLC-HRMS (Orbitrap) polyphenolic and capsaicinoid profiles proved to be adequate sample chemical descriptors for the characterization, classification, and authentication of paprika samples, according to both their PDO and production region and their flavor varieties. UHPLC-HRMS bioactive compound profiling using the TraceFinder^TM^ screening software v3.3. (Thermo Fisher Scientific) without the requirement of target compound quantitation through commercially available standards, was proposed with the aim of obtaining a feasible, simple, and cheaper methodology. For this purpose, two homemade accurate mass databases consisting of 53 polyphenolic compounds, and 12 capsaicinoid and capsinoid compounds, were employed.

Exploratory analysis through PCA and a classification study through PLS-DA, using the obtained UHPLC-HRMS polyphenolic profiles, showed good discrimination capabilities among the different paprika PDO and production regions, in general, with acceptable PLS-DA classification ratios. Additionally, perfect discrimination and authentication capabilities were also accomplished for the Murcia PDO and the Czech Republic flavor varieties. In contrast, only a slight discrimination was achieved for the three commercially available La Vera PDO flavor varieties.

The capability of the proposed methodology to address the La Vera PDO flavor variety classification and authentication clearly improved when UHPLC-HRMS polyphenolic profiles were combined with the UHPLC-HRMS capsaicinoid and capsinoid profiles. A perfect separation of the spicy flavor variety from the other two groups, as expected by the high presence of capsaicinoid-based compounds in the spicy group of samples, and an acceptable discrimination between both sweet and bittersweet La Vera PDO varieties were accomplished.

Therefore, the proposed target-UHPLC-HRMS polyphenolic and capsaicinoid profiling methodology resulted in a feasible, simple, and relatively cheap alternative to address the characterization, classification, and authentication of paprika samples.

## Figures and Tables

**Table 1:** TraceFinder^TM^ polyphenolic profiling report obtained for a sweet La Vera PDO paprika.

Target Name	+/−	Area	Formula	Expected *m/z*	Measured *m/z*	Delta *m/z*	Isotopic Pattern Score (%)
D-(-)-Quinic acid	−	7.19 × 10^8^	C7H12O6	191.0561	191.0558	−1.72	100
Ethyl gallate	−	5.99 × 10^6^	C9H10O5	197.0455	197.0452	−1.39	100
Polydatin	−	4.15 × 10^7^	C20H22O8	389.1242	389.1254	3.02	89
Syringic acid	−	5.99 × 10^6^	C9H10O5	197.0455	197.0452	−1.39	100
Gallic acid	−	2.35 × 10^7^	C7H6O5	169.0142	169.0137	−2.85	95
Arbutin	−	1.58 × 10^8^	C12H16O7	271.0823	271.0817	−2.23	100
3,4-Dihydroxybenzaldehyde	−	7.75 × 10^7^	C7H6O3	137.0244	137.0239	−3.68	80
4-Hydroxybenzoic acid	−	7.75 × 10^7^	C7H6O3	137.0244	137.0239	−3.68	80
Chlorogenic acid	−	3.91 × 10^7^	C16H18O9	353.0878	353.0870	−2.16	100
*p*-coumaric acid	−	4.52 × 10^7^	C9H8O3	163.0401	163.0395	−3.74	87
Caffeic acid	−	7.57 × 10^7^	C9H8O4	179.0350	179.0346	−2.28	100
Homovanillic acid	−	4.39 × 10^8^	C9H10O4	181.0506	181.0504	−1.36	100
Syringaldehyde	−	4.39 × 10^8^	C9H10O4	181.0506	181.0504	−1.36	100
Veratric acid	−	4.39 × 10^8^	C9H10O4	181.0506	181.0504	−1.36	100
Homogentisic acid	−	3.73 × 10^7^	C8H8O4	167.0350	167.0345	−2.87	94
Vanillic acid	−	3.73 × 10^7^	C8H8O4	167.0350	167.0345	−2.99	94
Trans-Cinnamic acid	−	1.79 × 10^7^	C9H8O2	147.0452	147.0446	−3.86	100
Umbelliferon	−	1.17 × 10^7^	C9H6O3	161.0244	161.0241	−1.90	99
Ferulic acid	−	4.52 × 10^7^	C10H10O4	193.0506	193.0504	−1.27	100
Rutin	−	4.86 × 10^7^	C27H30O16	609.1461	609.1454	−1.17	100
Taxifolin	−	2.01 × 10^6^	C15H12O7	303.0510	303.0505	−1.83	100
(+)-catechin	−	9.48 × 10^5^	C15H14O6	289.0718	289.0713	−1.63	91
Quercitrin hydrate	−	1.19 × 10^9^	C21H20O11	447.0933	447.0924	−2.07	100
Homoplantaginin	−	2.71 ×10^7^	C22H22O11	461.1089	461.1081	−1.75	91
Fisetin	−	1.41 × 10^8^	C15H10O6	285.0405	285.0397	−2.55	100
Quercetin	−	1.60 × 10^7^	C15H10O7	301.0354	301.0346	−2.74	90
Resveratrol	−	3.49 × 10^6^	C14H12O3	227.0714	227.0705	−3.92	89
Rosmanol	−	5.66 × 10^6^	C20H26O5	345.1707	345.1700	−2.17	100
Asiatic acid	−	2.59 × 10^6^	C30H48O5	487.3429	487.3419	−2.08	100
Cirsimaritin	−	1.95 × 10^6^	C17H14O6	313.0718	313.0710	−2.51	91
Carnosol	−	5.15 × 10^6^	C20H26O4	329.1758	329.1750	−2.37	86
Carnosic acid	−	2.41 × 10^6^	C20H28O4	331.1915	331.1909	−1.85	100
Ursolic acid	−	6.52 × 10^5^	C30H46O3	453.3374	453.3362	−2.55	100

## Supplementary Materials

The following are available online at https://www.mdpi.com/1420-3049/25/7/1623/s1, Table S1. Chemical structures of the studied capsaicinoids and capsinoids. Table S2. HRMS spectral data of the studied Capsaicinoids and Capsinoids. Figure S1. 3D plot of scores of PLS-DA model (LV1 vs. LV2 vs. LV3) when the UHPLC-HRMS polyphenolic profiles were employed as chemical descriptors of the analyzed paprika samples according to their production regions and flavor varieties.
