# Supplementary material for: Targeted UHPLC–HRMS (Orbitrap) Polyphenolic and Capsaicinoid Profiling for the Chemometric Characterization and Classification of Paprika with Protected Designation of Origin (PDO) Attributes
Source: Molecules. 2020 Apr 1;25(7):1623. doi: 10.3390/molecules25071623 (PMC7181276; doi:10.3390/molecules25071623)
Supplement: Supplementary file 1 [file molecules-25-01623-s001.pdf]

Supplementary Material

Targeted UHPLC-HRMS (Orbitrap) Polyphenolic and Capsaicinoid Profiling for the Chemometric Characterization and Classification of Paprika with Protected Designation of Origin (PDO) Attributes

Sergio Barbosa [1], Javier Saurina [1,2], Lluís Puignou [1,2] and Oscar Núñez [1,2,3,*]

[1] Department of Chemical Engineering and Analytical Chemistry, University of Barcelona, Martí i Franquès 1-11, E08028 Barcelona, Spain; sergiobarbosabarbero@hotmail.com (S.B.); lluis.puignou@ub.edu (L.P.), xavi.saurina@ub.edu (J.S.); oscar.nunez@ub.edu (O.N.)

[2] Research Institute in Food Nutrition and Food Safety, University of Barcelona, Recinte Torribera, Av. Prat de la Riba 171, Edifici de Recerca (Gaudí), Santa Coloma de Gramenet, E08921 Barcelona, Spain

[3] Serra Húnter Fellow, Generalitat de Catalunya, Rambla de Catalunya 19-21, E08007 Barcelona, Spain

* Correspondence: oscar.nunez@ub.edu; Tel.: +34-93-403-3706

Table 1. Chemical structures of the studied capsaicinoids and capsinoids.

Compounds	Formula	CAS number	Structure
		Capsaicinoids	
Capsaicin	$C_{18}H_{27}NO_3$	404-86-4	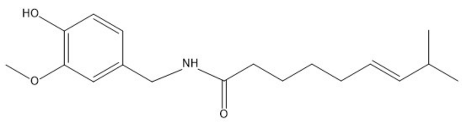
Dihydrocapsaicin	$C_{18}H_{29}NO_3$	19408-84-5	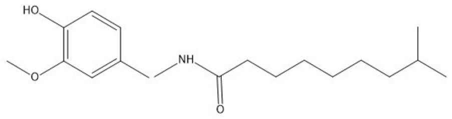
Homocapsaicin	$C_{19}H_{29}NO_3$	58493-48-4	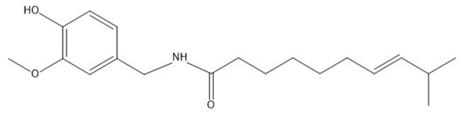
Homodihydrocapsaicin	$C_{19}H_{31}NO_3$	20279-06-5	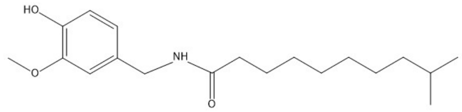
Nonivamide	$C_{17}H_{27}NO_3$	2444-46-4	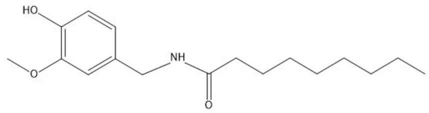

Norcapsaicin	$C_{17}H_{25}NO_3$	61229-08-1	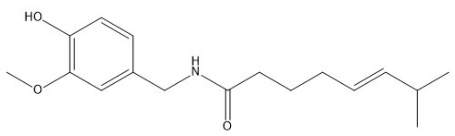
Nordihydrocapsaicin	$C_{17}H_{27}NO_3$	28789-35-7	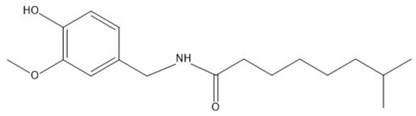
N-vanillyl decanamide	$C_{18}H_{29}NO_3$	31078-36-1	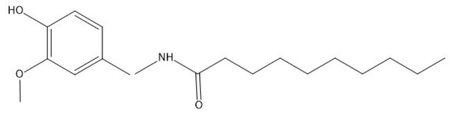
N-vanillyl octanamide	$C_{16}H_{25}NO_3$	58493-47-3	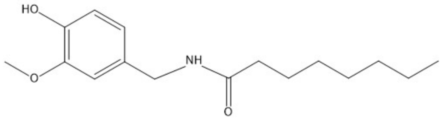
		Capsinoids	
Dihydrocapsiate	$C_{18}H_{28}O_4$	205687-03-2	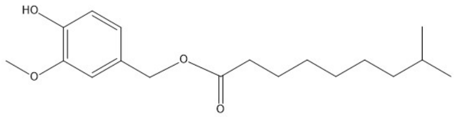
Capsiate	$C_{18}H_{26}O_4$	205687-01-0	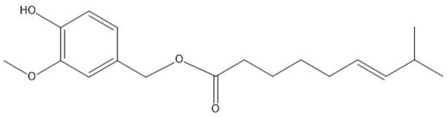
Nordihydrocapsiate	$C_{17}H_{26}O_4$	220012-53-3	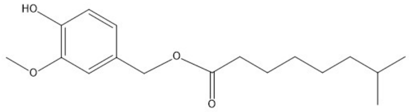

Table 2. HRMS spectral data of the studied Capsaicinoids and Capsinoids.

Compounds	Chemical formula	HRMS spectrum		
		[M-H]$^-$ *m/z* calculated value	[M-H]$^-$ *m/z* experimental value	Accurate mass error (ppm)
Capsaicinoids				
Capsaicin	$C_{18}H_{27}NO_3$	304.1918	304.1912	-1.972
Dihydrocapsaicin	$C_{18}H_{29}NO_3$	306.2075	306.2068	-2.286
Homocapsaicin	$C_{19}H_{29}NO_3$	318.2075	318.2069	-1.886
Homodihydrocapsaicin	$C_{19}H_{31}NO_3$	320.2231	320.2227	-1.249
Nonivamide	$C_{17}H_{27}NO_3$	292.1918	292.1913	-1.711
Norcapsaicin	$C_{17}H_{25}NO_3$	290.1762	290.1755	-2.412
Nordihydrocapsaicin	$C_{17}H_{27}NO_3$	292.1918	292.1913	-1.711
N-vanilly decanamide	$C_{18}H_{29}NO_3$	306.2075	306.2068	-2.286
N-vanilly octanamide	$C_{16}H_{25}NO_3$	278.1762	278.1755	-2.516
Capsinoids				
Dihydrocapsiate	$C_{18}H_{28}O_4$	307.1915	307.1907	-2.6042
Capsiate	$C_{18}H_{26}O_4$	305.1758	305.1751	-2.2938
Nordihydrocapsiate	$C_{17}H_{26}O_4$	293.1758	293.175	-2.7287

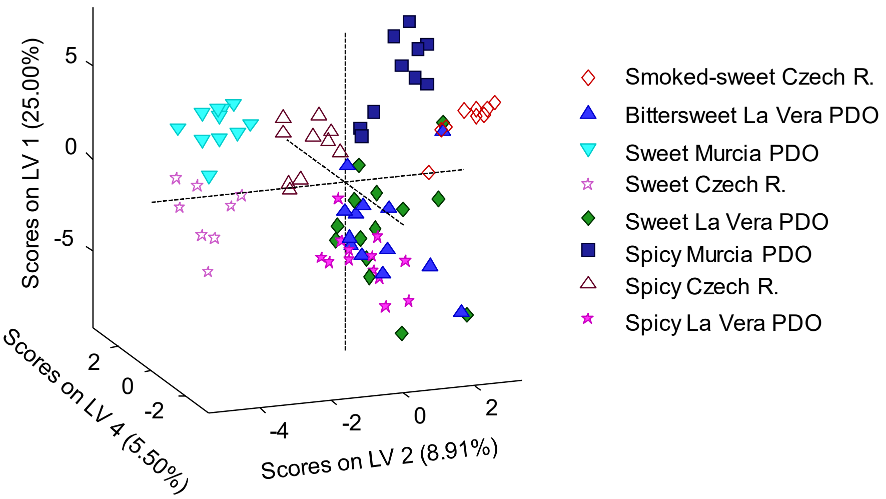

Figure 1. 3D plot of scores of PLS-DA model (LV1 vs. LV2 vs. LV3) when UHPLC-HRMS polyphenolic profiles were employed as chemical descriptors of the analyzed paprika samples according to their production regions and flavor varieties.
